# Scalable Exfoliation of Bulk MoS_2_ to Single- and Few-Layers Using Toroidal Taylor Vortices

**DOI:** 10.3390/nano8080587

**Published:** 2018-08-01

**Authors:** Vishakha Kaushik, Shunhe Wu, Hoyoung Jang, Je Kang, Kyunghoon Kim, Ji Won Suk

**Affiliations:** 1 School of Mechanical Engineering, Sungkyunkwan University, Suwon, Gyeonggi-do 16419, Korea; vishakhakaushik@gmail.com (V.K.); wushunhe@skku.edu (S.W.); kufjang@gmail.com (H.J.); jekang@skku.edu (J.K.); 2 SKKU Advanced Institute of Nanotechnology (SAINT), Sungkyunkwan University, Suwon, Gyeonggi-do 16419, Korea

**Keywords:** molybdenum disulfide, exfoliation, Taylor-Couette flow, continuous production

## Abstract

The production of a large amount of high-quality transition metal dichalcogenides is critical for their use in industrial applications. Here, we demonstrate the scalable exfoliation of bulk molybdenum disulfide (MoS_2_) powders into single- or few-layer nanosheets using the Taylor-Couette flow. The toroidal Taylor vortices generated in the Taylor-Couette flow provide efficient mixing and high shear stresses on the surfaces of materials, resulting in a more efficient exfoliation of the layered materials. The bulk MoS_2_ powders dispersed in *N*-methyl-2-pyrrolidone (NMP) were exfoliated with the Taylor-Couette flow by varying the process parameters, including the initial concentration of MoS_2_ in the NMP, rotation speed of the reactor, reaction time, and temperature. With a batch process at an optimal condition, half of the exfoliated MoS_2_ nanosheets were thinner than ~3 nm, corresponding to single to ~4 layers. The spectroscopic and microscopic analysis revealed that the exfoliated MoS_2_ nanosheets contained the same quality as the bulk powders without any contamination or modification. Furthermore, the continuous exfoliation of MoS_2_ was demonstrated by the Taylor-Couette flow reactor, which produced an exfoliated MoS_2_ solution with a concentration of ~0.102 mg/mL. This technique is a promising way for the scalable production of single- or few-layer MoS_2_ nanosheets without using hazardous intercalation materials.

## 1. Introduction

Two-dimensional (2D) nanomaterials such as graphene and transition metal dichalcogenides (TMDs) have captured widespread attention in recent years [1,2,3,4,5,6]. In contrast to semi-metallic graphene with a zero band gap, several TMDs are semiconductors with sizable band gaps [7,8]. For example, bulk molybdenum disulfide (MoS_2_) has an indirect band gap of 1.2 eV, while the monolayer MoS_2_ has a direct band gap of 1.8~1.9 eV [7]. Their layer-dependent and tunable band gap transition make the materials special for future electronics applications [9,10,11].

The large-scale production of TMDs is a crucial and essential step for their realization in industrial applications. Liquid-phase exfoliation is a promising means for producing high-quality ultra-thin nanosheets with a high throughput [12]. Lithium-based intercalation has been a classical exfoliation method for MoS_2_ [13]. During the liquid-phase exfoliation, two steps are involved; (i) initially, the foreign species intercalate between the adjacent layers, resulting in an increase of the interlayer spacing and a decrease of the interlayer interaction, and (ii) each layer is exfoliated by subsequent treatments using external forces such as sonication. However, the lithium-based exfoliation has several issues such as a long intercalation time, flammability of lithium in air, and tedious purifying steps, which are undesirable for an environmentally friendly and low-cost production in the industry [14]. Furthermore, the lithium intercalation induces the phase transformation of MoS_2_ from semiconducting 2H-MoS_2_ to metallic 1T-MoS_2_, requiring additional treatments to recover its semiconducting structure [15].

As an alternative to the lithium-based exfoliation, Coleman et al. have reported simple liquid-phase exfoliation of bulk MoS_2_ powders using sonication with common solvents [16]. The exfoliated MoS_2_ nanosheets had thicknesses of ~3 nm to ~12 nm corresponding to ~4 to ~17 layers, as the thickness of the monolayer MoS_2_ is ~0.7 nm. Although the sonication treatment is efficient to apply high energy, it may induce defects like tears and pinholes in exfoliated layers. In addition, it was difficult to obtain the monolayer MoS_2_ when they used the sonication treatment. Therefore, Coleman and his colleagues utilized strong shear forces to efficiently exfoliate the MoS_2_ after dispersing the MoS_2_ powders in aqueous surfactant solutions [17]. Even though the work demonstrated a potential of shear exfoliation on a large scale, the exfoliation is highly dependent on the surfactant concentration and the surfactant may need to be removed for further applications.

In order to avoid the use of intercalating agents and to improve the exfoliation efficiency, we demonstrate a facile exfoliation method using toroidal Taylor vortices in the Taylor-Couette flow. The Taylor-Couette flow is generated in a small gap between two concentric cylinders; the inner cylinder rotates while the outer one is fixed. If the shaft rotates at a higher rate than a critical point, the centrifugal force exceeds the viscous force and toroidal Taylor vortices are formed. The formation of these vortices in the annulus along with the cylinders generates high shear forces with homogeneous turbulent mixing [18]. Therefore, the Taylor-Couette flow has been used for enhancing the dispersion of particles and chemical reactions [19,20,21]. Recently, it has also been utilized in exfoliating graphite into graphene or graphene oxide nanosheets [22,23,24]. In this work, we applied the Taylor-Couette flow to exfoliate bulk MoS_2_ powders into single or few layers. Instead of using an organic intercalating agent, a common organic solvent was used for helping with the exfoliation and dispersion of the MoS_2_ nanosheets. Furthermore, the Taylor-Couette flow reaction was adapted for a continuous exfoliation process of MoS_2_ nanosheets, presenting a potential for scalable production.

## 2. Materials and Methods

### 2.1. Exfoliation of MoS_2_ Layers with Taylor-Couette Flow

Bulk MoS_2_ powders (CAS-804169, Sigma Aldrich, St. Louis, MO, USA) with lateral sizes ranging from sub micrometer to a few micrometers were initially grinded by a mortar and pestle, prior to exfoliation in the Taylor-Couette flow. A small amount of *N*-methyl-2-pyrrolidone (NMP, Dae-Jung Chemicals and Metals) was added into the powders and grinded for about 3 h. The shapes of the raw and grinded MoS_2_ powders are shown in Figure S1 (Supplementary Materials).

**Figure 1:**
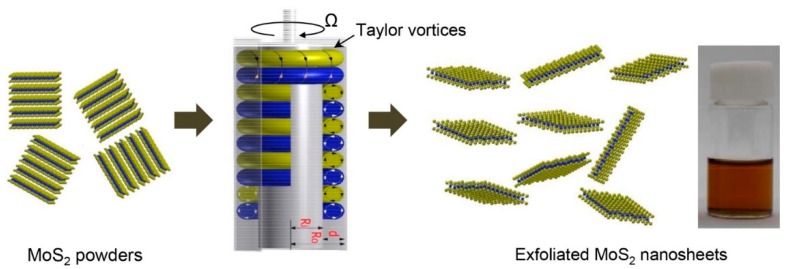
Schematic illustration of the exfoliation process of MoS_2_ using the Taylor-Couette flow.

The Taylor-Couette flow reactor (Laminar Co. Ltd., Seongnam, South Korea) consisted of two concentric cylinders with a small gap distance of 1.125 mm (Figure 1). The inner cylinder rotates while the outer cylinder is stationary. The gap between the inner shaft and inner wall of the outer jacket had a volume capacity of 12 mL and was surrounded with circulating hot water to control the reaction temperature. The grinded MoS_2_ powders mixed with NMP were fed into the gap of the reactor and the inner shaft was rotated for a given reaction time. After completing the exfoliation process, the MoS_2_ dispersed in the NMP was obtained and centrifuged at 1500 rpm for 30 min to remove the unexfoliated MoS_2_ flakes. The upper half of the centrifuged solution was decanted for collecting the exfoliated MoS_2_ nanosheets.

### 2.2. Characterization

The morphology of the exfoliated MoS_2_ nanosheets was observed by scanning electron microscopy (SEM, Jeol JSM-7600) after vacuum filtration with an anodic aluminum oxide (AAO) membrane. High-resolution transmission electron microscopy (TEM, Jeol ARF 200F, Tokyo, Japan) with CEOS Cs aberration correctors was used to investigate the atomic structures of the exfoliated nanosheets. The thicknesses of the exfoliated MoS_2_ nanosheets were characterized by atomic force microscopy (AFM, Seiko Instruments Inc., Chiba, Japan, SII-SPA-300HV) in a tapping mode after placing MoS_2_ nanosheets on a mechanically cleaved mica surface. The chemical structures of the exfoliated MoS_2_ were characterized with X-ray photoelectron microscopy (XPS, Thermo-Scientific, Waltham, MA, USA, ESCALAB-250 with a monochromated Al K_α_ radiation), Raman spectroscopy (Nanobase, with a 532 nm wavelength laser), and a UV-VIS spectrophotometer (Varian, Cary-5000 UV-vis-NIR, Palo Alto, CA, USA). For the XPS analysis, a survey scan was performed to identify the peaks of all of the elements, and calibration was done with respect to the C 1s peak at 285.0 eV. The peak deconvolution was done and all of the peaks were fit with Gaussian-Lorentzian functions after the subtraction of Shirley-type baselines. To estimate the concentration of the exfoliated MoS_2_ in a solution, the mass of the exfoliated nanosheets was measured with a microbalance, after vacuum-filtering the solution on an AAO membrane and drying at 60 °C for 5 h in a vacuum.

## 3. Results and Discussion

### 3.1. Exfoliation of Bulk MoS_2_ in NMP

As an exfoliation and dispersion solvent, NMP was selected for this work. For an efficient exfoliation of MoS_2_, the surface energy of the solvent needs to be matched with that of MoS_2_ in a thermodynamic point of view [16,25,26]. NMP has a surface tension (σ) of ~40 mJ/m^2^ and its surface energy (γ) is governed by γ = σ + TS_S_, where T is the temperature, S_S_ is the surface entropy (~0.1 mJ/K·m^2^), and TS_S_ = ~29 mJ/m^2^ for liquids at room temperature [26]. Thus, the surface energy of the NMP closely matches that of the MoS_2_ (~75 mJ/m^2^) [26]. However, considering only the surface energy (or surface tension) value is not sufficient for effective exfoliation, as it has been observed that many solvents having similar surface energies exhibited a worse exfoliation [16]. The surface tension can be divided into two components, polar and dispersive interactions. It has been found that the ratio of polar (σ^p^) to dispersive (σ^d^) components of the surface tension is more important for selecting a suitable solvent when exfoliating 2D materials [27,28]. The polar to dispersive component ratios (σ^p^/σ^d^) for NMP and MoS_2_ are 0.396 and 0.449, respectively [29]. Thus, NMP is a proper solvent for the exfoliation process of MoS_2_.

Prior to the exfoliation process in the Taylor-Couette flow, the bulk MoS_2_ powders were grinded with a small amount of NMP. The grinding process provided thinner MoS_2_ flakes with reduced lateral sizes, helping the subsequent exfoliation of MoS_2_ in the Talyor-Couette flow (Figure S1 in Supplementary Materials).

In the Taylor-Couette flow, the toroidal Taylor vortices are formed when the Taylor number (*T_a_*) exceeds the critical value of ~1700. The Taylor number is calculated by the following [24,30]:

(1) $$T_{a}=\frac{\Omega^{2}R_{i}{\left(R_{o}-R_{i}\right)}^{3}}{\nu^{2}},$$

where Ω is the angular velocity, *ν* is the kinematic viscosity, and *R_o_* and *R_i_* are the radii of the outer and inner cylinders, respectively. Based on the equation, the Taylor number is above the critical value even at a low rotation speed in this work, *T_a_* = ~1.25 × 10^4^ for 500 rpm, which confirms the formation of the Taylor vortices over the whole experiments. In addition, it has been known that the average wall shear rate in the Taylor-Couette flow increases with the increasing the angular velocity [31]. Thus, increasing the rotational speed of the inner cylinder in the Taylor-Couette flow reactor is expected to enhance the exfoliation efficiency. The wall shear stress in the Taylor-Couette flow can be estimated by using the computational fluid dynamics (CFD) simulation. The previous work reported that the maximum shear stress of the Taylor-Couette flow in NMP increased from 3.98 Pa at a rotation speed of 500 rpm to 26.91 Pa at 3000 rpm, where the reactor had a gap size of 2.5 mm and an inner shaft diameter of 52 mm [24]. Even though the reactor dimensions were different from those in our work, the CFD results showed that the shear stress in the flow is highly dependent on the rotation speed.

### 3.2. Parametric Study for Optimizing the Exfoliation Process

**Figure 2:**
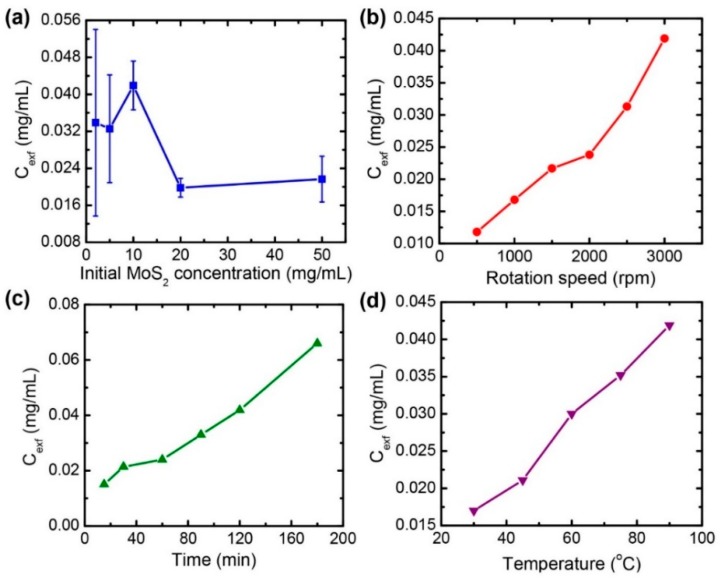
Change of the concentration of the exfoliated MoS_2_ nanosheets (C_exf_) as a function of (**a**) the initial MoS_2_ concentration, (**b**) the rotation speed of the inner cylinder shaft, (**c**) the reaction time, and (**d**) the reaction temperature.

Several factors can affect the exfoliation process with the Taylor-Couette flow. To find an optimal exfoliation condition for a higher concentration of exfoliated MoS_2_, four parameters were investigated, namely: the initial concentration of MoS_2_ in a solution, the reaction time, the rotation speed of the inner cylinder, and the reaction temperature. Firstly, the initial MoS_2_ concentration in NMP varied from 1 mg/mL to 50 mg/mL at a rotation speed of 3000 rpm, a reaction temperature of 90 °C, and a reaction time of 120 min. It is found that the maximum concentration of the exfoliated MoS_2_ was ~0.0419 mg/mL at the initial MoS_2_ concentration of 10 mg/mL (Figure 2a). However, further increasing the initial MoS_2_ concentration leads an adverse effect on the exfoliated quantity. It might be due to the increased viscosity of the MoS_2_ solution with more MoS_2_, resulting in lower shear forces in the Taylor-Couette flow. Thus, the optimum condition for the initial MoS_2_ solution concentration was 10 mg/mL, and we kept this for the other exfoliation experiments.

As discussed above, the local shear forces are highly dependent on the rotation speed in the Taylor-Couette flow. We varied the rotation speed of the inner cylinder from 500 rpm to 3000 rpm at a fixed reaction time of 120 min and a reaction temperature of 90 °C. It can be clearly noticed from Figure 2b that the concentration of the exfoliated MoS_2_ increases when increasing the rotation speed. The results confirm the presence of a higher shear rate and wall shear stress at a higher rotation speed in the Taylor-Couette flow, which is critical for the efficient exfoliation of MoS_2_. Therefore, we have chosen the rotation speed of 3000 rpm (the maximum speed of the reactor) for all of the experiments in this study.

Another important experimental parameter is the reaction time, because other existing exfoliation methods consume too much time to exfoliate the layers of their bulk materials. Thus, a method that can save a process time with efficient exfoliation results would be a promising choice for the scalable production of few-layer MoS_2_. Figure 2c shows the concentration change of the exfoliated MoS_2_ by varying the reaction time from 15 min to 180 min, while fixing the rotation speed (3000 rpm) and the reaction temperature (90 °C). It is observed that the concentration of the exfoliated MoS_2_ increases with the increasing the reaction time.

Also, the reaction time was varied from 30 °C to 90 °C by circulating water through the outside cylinder, while fixing the rotation speed (3000 rpm) and the reaction time (120 min). We observed that the concentration of the exfoliated MoS_2_ nanosheets increased when increasing the reaction temperature, as shown in Figure 2d. Generally, the viscosity of the liquid decreases with an increase in temperature. The decrease in the kinematic viscosity indicates the increase of the Taylor number, meaning an increased shear rate. Therefore, the elevated temperature helped the exfoliation of MoS_2_ in the Taylor-Couette flow. Furthermore, this may be partially due to the auto-oxidation of NMP at an elevated temperature [29]. It is known that NMP can be automatically oxidized at an elevated temperature with the presence of oxygen and water from the atmosphere. This leads to the formation of active intermediate species, such as radicals and hydroperoxide, which may oxidize the MoS_2_ edges and help the exfoliation of MoS_2_ [29].

### 3.3. Characterization of Exfoliated MoS_2_ Nanosheets

**Figure 3:**
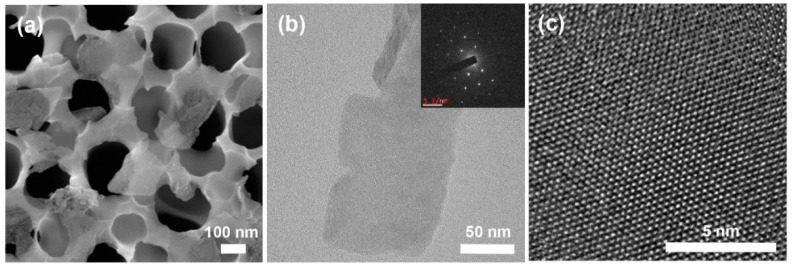
(**a**) SEM image of the exfoliated MoS_2_ nanosheets placed on an anodic aluminum oxide (AAO) membrane; (**b**) TEM image of the exfoliated MoS_2_ nanosheet; the inset shows the selected area electron diffraction (SAED) pattern for the MoS_2_ nanosheet; (**c**) High-resolution TEM image showing the single crystalline structure without defects.

Figure 3a shows a SEM image of the MoS_2_ nanosheets exfoliated at the optimized reaction condition. The exfoliated nanosheets were vacuum-filtered and placed on an AAO filter membrane. It can be clearly seen that the nanosheets are relatively thin compared to original MoS_2_ powders (Figure S1 in Supplementary Materials). The nanosheets are thin enough to see through the pore structures of the AAO membrane. The atomic structures of the exfoliated MoS_2_ were analyzed by TEM, as shown in Figure 3b,c. The nanosheets that appeared to be well exfoliated had single or few layers. Figure 3b shows a monolayer MoS_2_ nanosheet with a typical hexagonal pattern in a selected area electron diffraction (SAED). The high-resolution TEM images show the high-quality crystalline structures of MoS_2_ (Figure 3c), confirming that the Taylor-Couette flow reaction did not damage or destroy the intrinsic hexagonal structure of MoS_2_.

**Figure 4:**
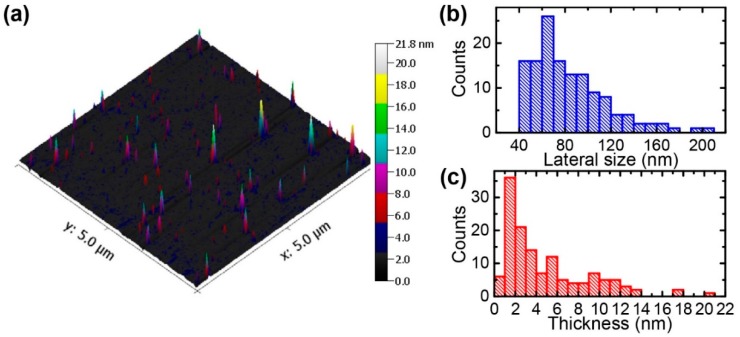
Atomic force microscopy (AFM) analysis of the exfoliated MoS_2_ nanosheets. (**a**) Topological image of the MoS_2_ nanosheets on mica; (**b**,**c**) Statistical distributions of the lateral sizes and thicknesses of the nanosheets obtained from (**a**).

The size and thickness of the exfoliated MoS_2_ nanosheets were investigated by AFM measurements, as shown in Figure 4. The mechanically cleaved mica was used as a substrate for the AFM samples, because of its atomically flat surface. Figure 4a shows a morphology image of the deposited MoS_2_ nanosheets; a scan in a smaller area is shown in Figure S2 (Supplementary Materials). Figure 4b shows the histograms for the corresponding thickness and lateral size of the sample. More than 130 nanosheets were measured and about 50% of the measured nanosheets were thinner than ~3 nm, indicating that the exfoliated MoS_2_ nanosheets were predominately thinner than ~4 layers, by assuming that monolayer thickness is ~0.7 nm. However, the exfoliated nanosheets show smaller lateral sizes compared to the electrochemically exfoliated MoS_2_ nanosheets [32].

**Figure 5:**
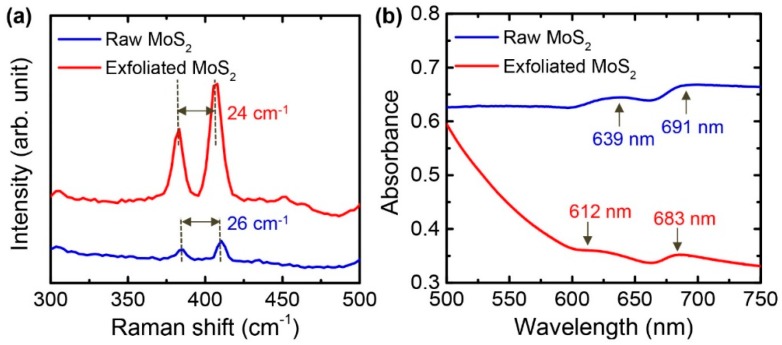
(**a**) Raman spectra of the bulk MoS_2_ powders and exfoliated MoS_2_ nanosheets; (**b**) UV-VIS spectra for the bulk MoS_2_ powders and exfoliated MoS_2_ nanosheets dispersed in *N*-methyl-2-pyrrolidone (NMP); solution concentrations were 10 mg/mL.

Figure 5a shows the typical Raman spectra of the bulk and exfoliated MoS_2_ nanosheets. Two characteristic peaks are found at 384 (E^1^_2g_) and 410 cm^−1^ (A_1g_) for the bulk MoS_2_. The E^1^_2g_ and A_1g_ are known to be related to in-plane opposite vibrations of sulfur/molybdenum atoms and out-of-plane vibrations of sulfur atoms, respectively [32,33]. As the bulk MoS_2_ was exfoliated to nanosheets, the E^1^_2g_ and A_1g_ peaks shifted to 382 and 406 cm^−1^, and the frequency difference between the two peaks decreased from 26 cm^−1^ to 24 cm^−1^. The change of positions and the frequency difference of the E^1^_2g_ and A_1g_ peaks are attributed to the change of the MoS_2_ layer numbers [33]. The frequency difference of 24 cm^−1^ of the exfoliated MoS_2_ nanosheets corresponds to roughly 4–5 layers [33].

A dispersion of the MoS_2_ flakes in NMP was characterized by UV-VIS absorption spectroscopy (Figure 5b). The exfoliated MoS_2_ nanosheets show two excitonic peaks at 612 nm and 683 nm, while the peaks for the bulk MoS_2_ are positioned at 639 nm and 691 nm. These excitonic peaks with an energy separation are due to the spin-orbital splitting of the valence band at the K-point of the Brillouin zone and the shift of the peaks is ascribed to the decrease of the layer number of MoS_2_ [34]. Therefore, the reaction in the Talyor-Couette flow exfoliated the bulk MoS_2_ and decreased its layer numbers.

**Figure 6:**
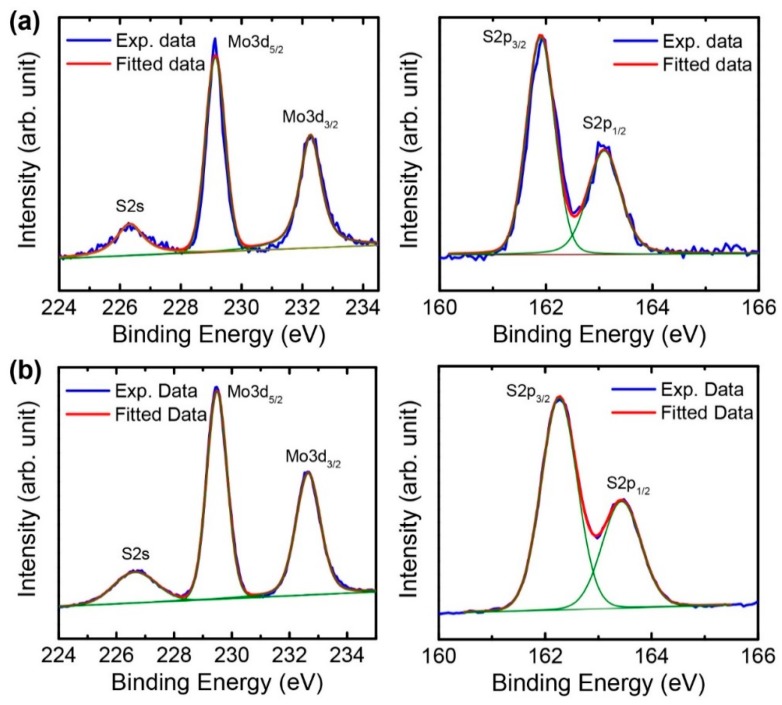
XPS spectra of (**a**) the bulk MoS_2_ powders and (**b**) exfoliated MoS_2_ nanosheets.

The chemical nature of the exfoliated MoS_2_ nanosheets was investigated using X-ray photoelectron spectroscopy (XPS). Figure 6 shows the XPS spectra of the experimental and fitted data for the bulk and exfoliated MoS_2_ samples. For the exfoliated MoS_2_ nanosheets, there are peaks for Mo 3d_5/2_ and Mo 3d_3/2_ at 229.4 and 232.6 eV, respectively, and additional peaks for S 2p_3/2_ and S 2p_1/2_ at 162.2 and 163.4 eV, respectively [32]. For the bulk MoS_2_, the peaks for Mo 3d_5/2_, Mo 3d_3/2_, S 2p_3/2_, and S 2p_1/2_ are positioned at 229.1, 232.3, 161.9, and 163.0 eV, respectively. The peaks for Mo and S in the XPS spectra of the exfoliated nanosheets are very similar to those of the bulk powders, indicating that the exfoliation process in the Taylor-Couette flow did not alter the original chemical structures of the bulk MoS_2_. In addition, the peak positions and shapes for the Mo and S of the exfoliated nanosheets are the same as those of 2H-MoS_2_ nanosheets, which were obtained by chemical exfoliation and subsequent thermal annealing [35]. This confirms that the exfoliated nanosheets kept semiconducting the 2H-MoS_2_ phase after the exfoliation process.

### 3.4. Continuous Production of Exfoliated MoS_2_

**Figure 7:**
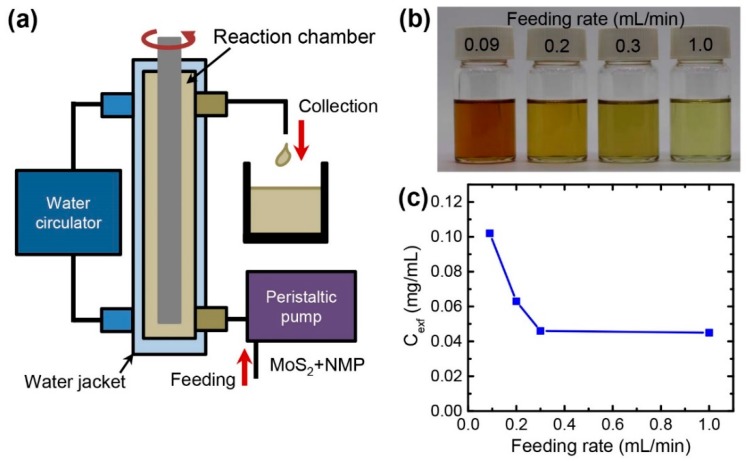
Continuous exfoliation of MoS_2_ using the Taylor-Couette flow. (**a**) Schematic illustration of the continuous process; (**b**) Color and (**c**) concentration change of the exfoliated MoS_2_ nanosheets as a function of the feeding rate during the continuous process.

In order to achieve a high-throughput production of the few-layer MoS_2_ nanosheets, the Taylor-Couette flow reactor was adapted for a continuous flow process. Figure 7a shows the schematic illustration of the continuous flow method. In this method, a MoS_2_ solution was continuously fed into the cylinder gap through the bottom of the reactor using a peristaltic pump with a constant flow rate. The inner cylinder was continuously rotating during the reaction and the excess of the solution was collected in the flask and again recirculated inside the reactor. In the whole process, the MoS_2_ dispersion was continuously sheared between the rotating inner and the stationary outer shaft of the reactor. The optimal conditions (initial MoS_2_ concentration = 10 mg/mL, rotation speed = 3000 rpm, and reaction temperature = 90 °C) were used for this continuous exfoliation.

Figure 7b,c shows the change of the concentration of the exfoliated MoS_2_ nanosheets after the reactions for 2 h. The slow feeding of the MoS_2_ solution generated a higher concentration of the exfoliated MoS_2_ solution. Interestingly, the maximum concentration of the exfoliated nanosheets was 0.102 mg/mL at the lowest feeding rate of 0.09 mL/min, which was higher compared to the nanosheets exfoliated in a batch process (0.0419 mg/mL). In this work, the flow reactor is vertically positioned. Thus, the bulk MoS_2_ powders could sink on the bottom of the reactor by gravity when they were exfoliated in a batch process. For the continuous exfoliation, the liquid could push up the bulk MoS_2_ powders and might induce a better dispersion and more shear interactions during the process, resulting in more exfoliation. However, with increasing the feeding rate, the MoS_2_ particles might pass through the Taylor vortices with reduced shear interactions, providing a low exfoliation efficiency. 

There have been several reports on the high-yield exfoliation of MoS_2_. For example, lithium intercalation and the subsequent sonication treatments generated thin MoS_2_ layers in a 92% yield [36]. However, the use of lithium may not be desirable for the environmentally friendly and low-cost production of MoS_2_ nanosheets because of several issues related to lithium, such as long intercalation time, flammability in air, and purification. Thus, a simple method using shear exfoliation in common solvents has been demonstrated for graphene with a concentration of ~0.3 mg/mL in a below 0.1% yield [37]. Our work demonstrated MoS_2_ exfoliation into single and few layers in reasonably high yield compared to the shear exfoliation of graphite in NMP. Thus, this continuous method using the Taylor-Couette flow leads to a more productive and stable exfoliation process for MoS_2_ and other 2D materials.

## 4. Conclusions

In this study, a facile and efficient method was presented for the exfoliation of bulk MoS_2_ layered crystals into a few layers. The toroidal Taylor vortices in the Taylor-Couette flow generated strong shear stress on MoS_2_, resulting in an efficient exfoliation in NMP. We found that half of the exfoliated nanosheets were thinner than ~3 nm (about ~4 layers) and the atomic and chemical characteristics were not altered by the exfoliation process. In addition, a continuous production system using the Taylor-Couette flow was demonstrated for exfoliating MoS_2_. The maximum concentration of the exfoliated nanosheets was ~0.102 mg/mL for the 2-h process. This method has potential for scalable production of few-layer MoS_2_ nanosheets, which is critical for various device applications in the future.

## Supplementary Materials

The following are available online at http://www.mdpi.com/2079-4991/8/8/587/s1, Figure S1: SEM images of raw and grinded MoS_2_ powders, Figure S2: AFM images of the exfoliated MoS_2_ nanosheets.

## References

[1] Butler S.Z., Hollen S.M., Cao L., Cui Y., Gupta J.A., Gutiérrez H.R., Heinz T.F., Hong S.S., Huang J., Ismach A.F. (2013). Progress, challenges, and opportunities in two-dimensional materials beyond graphene. ACS Nano.

[2] Chhowalla M., Liu Z., Zhang H. (2015). Two-dimensional transition metal dichalcogenide (tmd) nanosheets. Chem. Soc. Rev..

[3] Choi W., Choudhary N., Han G.H., Park J., Akinwande D., Lee Y.H. (2017). Recent development of two-dimensional transition metal dichalcogenides and their applications. Mater. Today.

[4] Duan X., Wang C., Pan A., Yu R., Duan X. (2015). Two-dimensional transition metal dichalcogenides as atomically thin semiconductors: Opportunities and challenges. Chem. Soc. Rev..

[5] Tedstone A.A., Lewis D.J., O’Brien P. (2016). Synthesis, properties, and applications of transition metal-doped layered transition metal dichalcogenides. Chem. Mater..

[6] Lee J.Y., Shin J.H., Lee G.H., Lee C.H. (2016). Two-dimensional semiconductor optoelectronics based on van der waals heterostructures. Nanomaterials.

[7] Mak K.F., Lee C., Hone J., Shan J., Heinz T.F. (2010). Atomically thin MoS_2_: A new direct-gap semiconductor. Phys. Rev. Lett..

[8] Splendiani A., Sun L., Zhang Y.B., Li T.S., Kim J., Chim C.Y., Galli G., Wang F. (2010). Emerging photoluminescence in monolayer MoS_2_. Nano Lett..

[9] Kang M., Kim B., Ryu S.H., Jung S.W., Kim J., Moreschini L., Jozwiak C., Rotenberg E., Bostwick A., Kim K.S. (2017). Universal mechanism of band-gap engineering in transition-metal dichalcogenides. Nano Lett..

[10] Ouyang B., Mi Z., Song J. (2016). Bandgap transition of 2h transition metal dichalcogenides: Predictive tuning via inherent interface coupling and strain. J. Phys. Chem. C.

[11] Su X., Ju W., Zhang R., Guo C., Yong Y., Cui H., Li X. (2016). Band gap modulation of transition-metal dichalcogenide mx2 nanosheets by in-plane strain. Physica E.

[12] Lin Z., Karthik P.S., Hada M., Nishikawa T., Hayashi Y. (2017). Simple technique of exfoliation and dispersion of multilayer graphene from natural graphite by ozone-assisted sonication. Nanomaterials.

[13] Xiong F., Wang H., Liu X., Sun J., Brongersma M., Pop E., Cui Y. (2015). Li intercalation in MoS_2_: In situ observation of its dynamics and tuning optical and electrical properties. Nano Lett..

[14] Forsberg V., Zhang R.Y., Backstrom J., Dahlstrom C., Andres B., Norgren M., Andersson M., Hummelgard M., Olin H. (2016). Exfoliated MoS_2_ in water without additives. PLoS ONE.

[15] Fan X.B., Xu P.T., Zhou D.K., Sun Y.F., Li Y.G.C., Nguyen M.A.T., Terrones M., Mallouk T.E. (2015). Fast and efficient preparation of exfoliated 2h MoS_2_ nanosheets by sonication-assisted lithium intercalation and infrared laser-induced 1t to 2h phase reversion. Nano Lett..

[16] Coleman J.N., Lotya M., O’Neill A., Bergin S.D., King P.J., Khan U., Young K., Gaucher A., De S., Smith R.J. (2011). Two-dimensional nanosheets produced by liquid exfoliation of layered materials. Science.

[17] Varrla E., Backes C., Paton K.R., Harvey A., Gholamvand Z., McCauley J., Coleman J.N. (2015). Large-scale production of size-controlled MoS_2_ nanosheets by shear exfoliation. Chem. Mater..

[18] Liu Z., Jin T., Kind M. (2013). Continuous polymerization of methyl methacrylate in a taylor-couette reactor. I. Influence of fluid dynamics on monomer conversion. Polym. Eng. Sci..

[19] Thai D.K., Mayra Q.-P., Kim W.-S. (2015). Agglomeration of ni-rich hydroxide crystals in taylor vortex flow. Powder Technol..

[20] Kim M., Park K.J., Lee K.U., Kim M.J., Kim W.-S., Kwon O.J., Kim J.J. (2014). Preparation of black pigment with the couette-taylor vortex for electrophoretic displays. Chem. Eng. Sci..

[21] Sanchez Fellay L., Vanni M. (2012). The effect of flow configuration on hydrodynamic stresses and dispersion of low density rigid aggregates. J. Colloid Interface Sci..

[22] Park W.K., Kim H., Kim T., Kim Y., Yoo S., Kim S., Yoon D.H., Yang W.S. (2015). Facile synthesis of graphene oxide in a couette-taylor flow reactor. Carbon.

[23] Park W.K., Yoon Y., Kim S., Choi S.Y., Yoo S., Do Y., Jung S., Yoon D.H., Park H., Yang W.S. (2017). Toward green synthesis of graphene oxide using recycled sulfuric acid via couette-taylor flow. ACS Omega.

[24] Tran T.S., Park S.J., Yoo S.S., Lee T.-R., Kim T. (2016). High shear-induced exfoliation of graphite into high quality graphene by taylor-couette flow. RSC Adv..

[25] Hernandez Y., Nicolosi V., Lotya M., Blighe F.M., Sun Z.Y., De S., McGovern I.T., Holland B., Byrne M., Gun’ko Y.K. (2008). High-yield production of graphene by liquid-phase exfoliation of graphite. Nat. Nanotechnol..

[26] Cunningham G., Lotya M., Cucinotta C.S., Sanvito S., Bergin S.D., Menzel R., Shaffer M.S.P., Coleman J.N. (2012). Solvent exfoliation of transition metal dichalcogenides: Dispersibility of exfoliated nanosheets varies only weakly between compounds. ACS Nano.

[27] Shen J., He Y., Wu J., Gao C., Keyshar K., Zhang X., Yang Y., Ye M., Vajtai R., Lou J. (2015). Liquid phase exfoliation of two-dimensional materials by directly probing and matching surface tension components. Nano Lett..

[28] Shen J., Wu J., Wang M., Dong P., Xu J., Li X., Zhang X., Yuan J., Wang X., Ye M. (2016). Surface tension components based selection of cosolvents for efficient liquid phase exfoliation of 2d materials. Small.

[29] Jawaid A., Nepal D., Park K., Jespersen M., Qualley A., Mirau P., Drummy L.F., Vaia R.A. (2016). Mechanism for liquid phase exfoliation of MoS_2_. Chem. Mater..

[30] White F.M. (2011). Fluid Mechanics.

[31] Dumont E., Fayolle F., Sobolik V., Legrand J. (2002). Wall shear rate in the taylor-couette-poiseuille flow at low axial reynolds number. Int. J. Heat Mass Trans..

[32] Liu N., Kim P., Kim J.H., Ye J.H., Kim S., Lee C.J. (2014). Large-area atomically thin MoS_2_ nanosheets prepared using electrochemical exfoliation. ACS Nano.

[33] Li H., Zhang Q., Yap C.C.R., Tay B.K., Edwin T.H.T., Olivier A., Baillargeat D. (2012). From bulk to monolayer MoS_2_: Evolution of Raman scattering. Adv. Funct. Mater..

[34] Nguyen T.P., Sohn W., Oh J.H., Jang H.W., Kim S.Y. (2016). Size-dependent properties of two-dimensional MoS_2_ and WS_2_. J. Phys. Chem. C.

[35] Eda G., Yamaguchi H., Voiry D., Fujita T., Chen M., Chhowalla M. (2011). Photoluminescence from chemically exfoliated MoS_2_. Nano Lett..

[36] Zeng Z.Y., Yin Z.Y., Huang X., Li H., He Q.Y., Lu G., Boey F., Zhang H. (2011). Single-layer semiconducting nanosheets: High-yield preparation and device fabrication. Angew. Chem. Int. Ed..

[37] Paton K.R., Varrla E., Backes C., Smith R.J., Khan U., O’Neill A., Boland C., Lotya M., Istrate O.M., King P. (2014). Scalable production of large quantities of defect-free few-layer graphene by shear exfoliation in liquids. Nat. Mater..

