# Supplementary material for: Scalable Exfoliation of Bulk MoS2 to Single- and Few-Layers Using Toroidal Taylor Vortices
Source: Nanomaterials (Basel). 2018 Aug 1;8(8):587. doi: 10.3390/nano8080587 (PMC6116258; doi:10.3390/nano8080587)
Supplement: Supplementary file 1 [file nanomaterials-08-00587-s001.pdf]

Supporting Materials

Scalable Exfoliation of Bulk MoS_2 to Single- and Few-Layers Using Toroidal Taylor Vortices

Vishakha Kaushik[1,†], Shunhe Wu[1,†], Hoyoung Jang[1], Je Kang[1], Kyunghoon Kim[1,] and Ji Won Suk[1,2,*]*

[1] School of Mechanical Engineering, Sungkyunkwan University, Suwon, Gyeonggi-do 16419, Republic of Korea

[2] SKKU Advanced Institute of Nanotechnology, Sungkyunkwan University, Suwon, Gyeonggi-do 16419, Republic of Korea

[†]These authors contributed equally to this work.

*Corresponding authors: kenkim@skku.edu (K. Kim) and jwsuk@skku.edu (J. W. Suk)

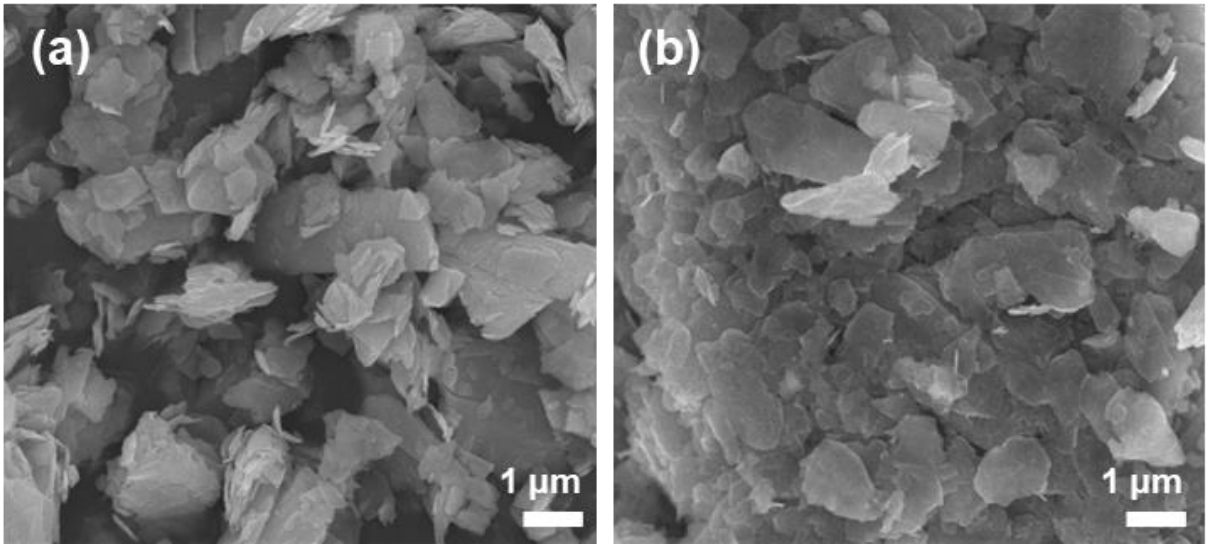

Figure S1. SEM images of (a) raw and (b) grinded MoS_2 powders.

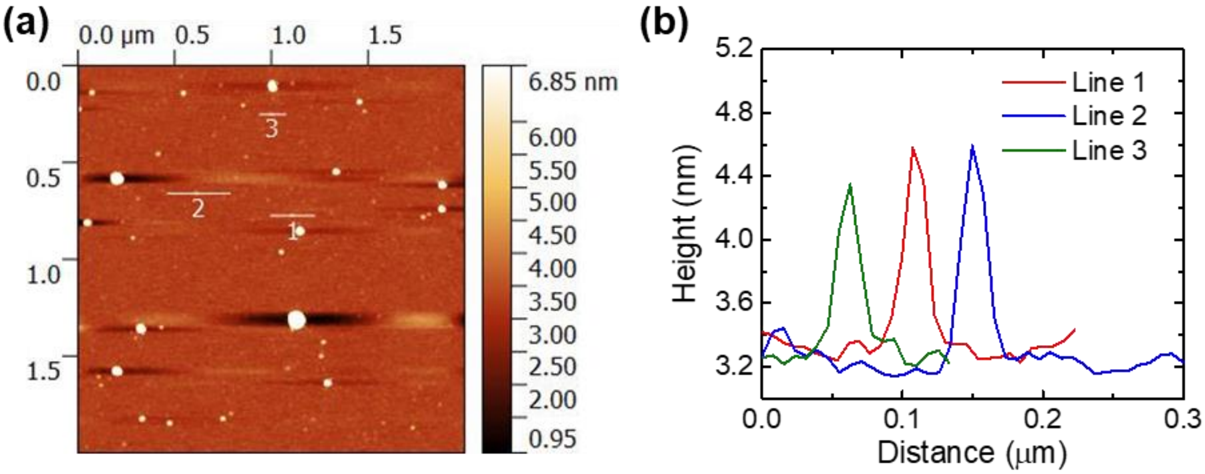

Figure S2. AFM analysis of the exfoliated MoS_2 nanosheets in a scan area of 2 µm × 2 µm. (a) Topological image. (b) Line profiles obtained from (a).
